# The smallest known species of Afrotropical *Scolytoplatypus* Schaufuss (Curculionidae, Scolytinae) – with unique features and an isolated phylogenetic position

**DOI:** 10.3897/zookeys.749.24199

**Published:** 2018-04-10

**Authors:** Bjarte H. Jordal

**Affiliations:** 1 Natural History Museum, The University Museum, University of Bergen, NO-5007 Bergen, Norway

**Keywords:** Afrotropics, ambrosia beetle, molecular phylogeny, Scolytoplatypodini

## Abstract

Recent flight intercept trapping in Gabon provided four female specimens of a new species of *Scolytoplatypus* Schaufuss with several unusual features. It is the smallest known Afrotropical species found to date (1.6 mm long), it has unusually long antennal clubs, and some characters show resemblance to small Asian species or to the Malagasy genus *Remansus* Jordal. Genetic data from four genes nevertheless place this species as the sister lineage to all other Afrotropical species where it forms an isolated position corresponding to deviant morphological features.

## Introduction

Species in the tribe Scolytoplatypodini are ambrosia beetles which cultivate fungi in wood tunnels as the only food source for larvae and adults. They are mainly old world tropical in distribution, with a few species found in temperate areas of Japan to India. Most species in the tribe are found in Asia with 29 known species (Beaver and Gebhardt 2006; Knížek 2008), whereas 11 or 12 are known in Africa (Browne 1971; Schedl 1975), and seven in Madagascar (Jordal 2013)

*Scolytoplatypus* Schaufuss has previously been regarded as a morphologically homogeneous genus. However, recent work has pointed out considerable variation in crucial anatomical parts such as the shape of the scutellum and the protibiae (Jordal 2013), or variation in sexual dimorphism across continents (Beaver and Gebhardt 2006). This led to the erection of a new genus *Remansus* Jordal and phylogenetic analyses documented deep divergence between this genus and *Scolytoplatypus*, and between Asian and African species. African species form a largely coherent group with rather few large differences between the species known to date.

An undescribed species with several unusual and intermediate features was recently collected in Gabon. DNA data clearly associate this species with the Afrotropical clade, and phylogenetic analyses indicate a rather isolated position of the species.

## Materials and methods

Samples were collected by flight intercept traps baited with vittatol and ipsenol lures in the Ipassa National Park, Gabon. Specimens were compared to types and co-types of most Afrotropical species in the Natural History Museum of Vienna, and some superficially similar Asian species.

DNA was extracted from a specimen using the Qiagen DNEasy kit. Amplification of four gene fragments (COI, EF1α, CAD, 28S) was made by PCR, using primers and cycling conditions described previously (Jordal et al. 2011). Concatenated DNA sequence data from Jordal (2013) were analysed in MrBayes v. 3.2.6 (Ronquist et al., 2012). Partitions were based on nucleotide positions per gene, or nucleotide positions combined, or by gene. Models were estimated in MrModeltest, selecting a GTR+G+I for each partition. 10 million generations were run, with 25% of the generations as burn-in. Stationarity was obtained after 500,000 generations and runs with PSRF close to 1.0 and standard deviation of split frequencies below 0.05 were accepted.

## Results

### Scolytoplatypus unipilus

Taxon classificationAnimaliaColepteraCurculionidae

Jordal sp. n.

http://zoobank.org/592D85B6-195F-4B4E-B2A6-23596B98BC73

Figs 1–4

#### Type material examined.

Holotype, female: Gabon: Ivindo National Park, Ipassa, 6 km W. Makokou. GIS: 0.512, 12.802, #23 vittatol trap. Paratypes (2): same data as holotype, except one taken from Ipsenol trap. The holotype and two paratypes (“ZMBN/ENTScol4942 – ZMBN/ENTScol4944”) are deposited in the University Museum of Bergen (ZMBN).

#### Diagnosis, female.

Typical female *Scolytoplatypus* with broad protibiae with transverse rows of granules and rugae, an anteromedian mycangial pore on pronotum, and a depressed triangular scutellum. Distinguished from all species in the genus by the unusually long antennal club, further from all African and Malagasy species by the small size (1.7 vs. >2.3 mm), the lack of striae on elytral declivity (and disk), by the undivided, simple setae on the metanepisternum, and the rounded hind corners of the pronotum.

**Figures 1–4.**
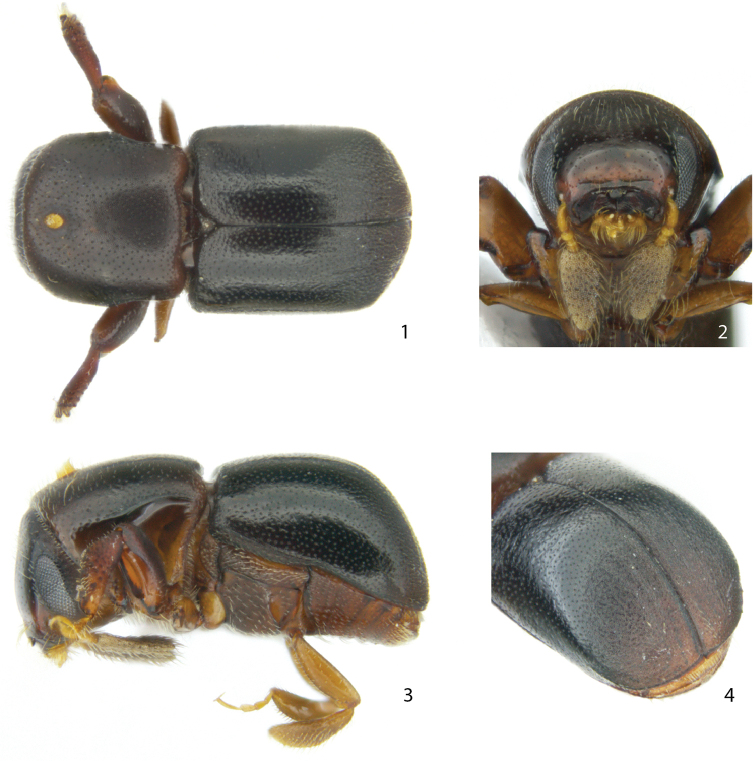
Habitus, head and elytral declivity of *Scolytoplatypus unipilus* sp. n.

#### Description, female.

*Length* 1.6–1.7 mm, 2.0 × longer than wide; *colour* dark brown to black, ventral side and legs brown.

*Head*. Eyes separated above by 3.9 × their width. Frons generally convex, slightly flattened on upper half, rounded below, with a transverse, broad, impression just above epistoma; surface smooth and shiny on lower half, reticulated and dull above, with small shallow punctures separated by 2–4 × their diameter. Vestiture consisting of scattered, short, fine setae mainly in reticulated area on upper half. Antennal club 3 × longer than funicle, densely covered by very short scale-like setae and fewer and much longer fine setae. Funiculus 5-segmented.

*Pronotum* 0.9 × as long as wide, sides subparallel on anterior half, constricted on posterior half, 0.9 × as wide as anterior part; surface finely reticulated with shallow punctures spaced by 1–2 × their diameter; pronotal vestiture consisting of fine short setae arising from punctures, a few longer setae scattered close to anterior margin. Mycangial pore slightly elliptical, with long yellow setae emerging, center of pore located on anterior fifth.

*Elytra* 1.1 × longer than wide, 1.3–1.4 × longer than pronotum; basal area notched for depressed triangular scutellum; sides of elytra straight, broadly rounded behind; striae not indicated, punctures confused, spaced on disc by 1–2 × their diameter; declivity finely rugose, strongly reticulated. Interstriae 10 weakly elevated to level of ventrite 1. Vestiture consisting of minute setae on declivity.

*Legs*. Procoxae separated by width of antennal club. Mesocoxae separated by width of a mesocoxa. Protibial shape typical for genus.

*Ventral vestiture.* Metanepisternum with relatively few, fine, simple setae.

#### Male.

Not known.

#### Molecular data.

**Figure 5.**
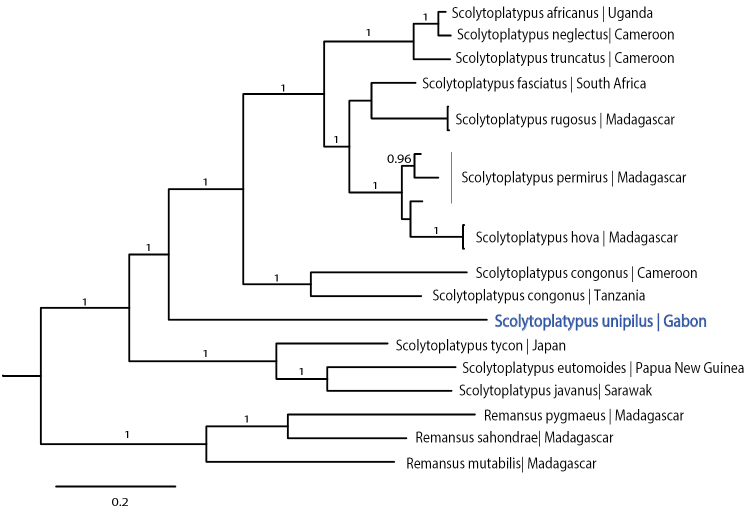
Tree topology (excluding outgroups) resulting from all Bayesian analyses (PSRF = 1.0, sd = 0.003) and the parsimony analysis (L = 3503, CI = 0.48, RI = 0.54), of four gene fragments.

Phylogenetic analysis based on four genes resulted in a fully resolved tree topology (Fig. 5). Different partition schemes and model selection had no influence on tree topology. *Scolytoplatypus unipilus* formed a maximally supported sister lineage to all other African and Malagasy species in the genus, and yet clearly separate from the Asian species. GeneBank accession numbers: COI, MG979488; EF1a, MG979489; CAD, MG979490; 28S, MG980072.

#### Etymology.

The Latin name *unipilus* is composed of the masculine adjective *unus* in its form uni-, meaning one, and the masculine noun *pilus*, meaning hair, referring to the simple, single, hair-like setae on the metanepisternum and metasternum.

#### Distribution and biology.

Only known from the type locality in Gabon. All specimens were collected in black flight intercept traps baited with vittatol (3) or ipsenol (1) lures.

### Key to females of African *Scolytoplatypus* species groups

**Table:** 

1	Antennal club as long as the eye, hind corners of pronotum rounded, setae on metanepisternum simple, not divided, female size 1.6-1.7 mm long	***S. unipilus* Jordal, sp. n.**
–	Antennal club at most 0.7 × as long as the eye, hind corners of pronotum acutely pointed laterally, setae on metanepisternum bifid, trifid or plumose, female size >2.3 mm long	**2**
2	Scutellum flush with elytra	***S. congonus* group** (*S. congonus* Schedl, *S. kivuensis* Schedl)
–	Scutellum depressed, narrowly elongated	3
3	Profemur with a dorsal spine near its distal end	***S. africanus* group** (*S. africanus* Eggers, *S. neglectus* Schedl, *S. occidentalis* Browne, *S. truncatus* Browne)
–	Profemur smooth, without dorsal spine	**4**
4	Sutural apex of elytra emarginated, notched	***S. armatus* group** (*S. armatus* Eggers, *S. eichelbaumi* Hagedorn)
–	Apex of elytra evenly rounded	**5**
5	Vestiture on declivity consisting of white scale-like setae	***S. uter* Schedl**
–	Declivity glabrous or with very fine setae	***S. fasciatus* group** (*S. fasciatus* Hagedorn, *S. opacicollis* Eggers, *S. obtectus* Schedl)

## Discussion

*Scolytoplatypus* is a very characteristic genus of ambrosia beetles, and even the smallest of the known species are larger than the average wood boring beetle. Nevertheless new species are being discovered and described after rather limited collecting efforts (Beaver and Gebhardt 2006; Browne 1971; Jordal 2013; Knížek 2008). This indicates quite strongly that many more species remain to be discovered.

It is interesting that recent field collections have revealed scolytoplatypodine taxa which are unique by having an isolated phylogenetic position. The genus *Remansus* was discovered only after collecting several new species in Madagascar (Jordal 2013). Likewise, the new species *S. unipilus* is the sister lineage to all other African species (Fig. 5) and shows several intermediate morphological traits. This taxon is, therefore, crucial to understand the evolution of the genus.

## Supplementary Material

XML Treatment for Scolytoplatypus unipilus
